# Comparison of efficiency and time to regeneration of *Agrobacterium*-mediated transformation methods in *Medicago truncatula*

**DOI:** 10.1186/s13007-019-0404-1

**Published:** 2019-02-28

**Authors:** Li Wen, Yuanling Chen, Elise Schnabel, Ashley Crook, Julia Frugoli

**Affiliations:** 1 Department of Genetics and Biochemistry, Clemson University, Clemson, USA; 2 Department of Food and Biological Engineering, Changsha University of Science and Technology, Changsha, People’s Republic of China; 3 College of Life Sciences, South China Agricultural University, Guangzhou, People’s Republic of China; 4 Department of Biology, University of North Carolina at Chapel Hill, Chapel Hill, NC 27599 USA

**Keywords:** *Medicago truncatula*, Tissue culture regeneration, Root explants, *crn* Mutant, Cytokinin reporter, R108, A17

## Abstract

**Background:**

Tissue culture transformation of plants has an element of art to it, with protocols passed on between labs but often not directly compared. As *Medicago truncatula* has become popular as a model system for legumes, rapid transformation is critical, and many protocols exist, with varying results.

**Results:**

The *M. truncatula* ecotypes, R108 and A17, were utilized to compare the effect of a modification to a previously used protocol based on shoot explants on the percentage of transformed plants produced from calli. This percentage was then compared to that of two additional transformation protocols based on root explants in the R108 ecotype. Variations in embryonic tissue sources, media components, time for transformation, and vectors were analyzed.

**Conclusions:**

While no A17 transgenic plants were obtained, transgenic plantlets from the R108 ecotype were produced in as little as 4 months with a comparison of the two widely studied ecotypes under a single set of conditions. While the protocols tested gave similar results in percentage of transformed plants produced, considerations of labor and time to transgenics that vary between the root explant protocols tested were discovered. These considerations may influence which protocol to choose for introducing a single transgene versus creating lines with multiple mutations utilizing a CRISPR/Cas9 construct.

**Electronic supplementary material:**

The online version of this article (10.1186/s13007-019-0404-1) contains supplementary material, which is available to authorized users.

## Background

Plant transformation is one of the biggest bottlenecks to progress in crop plant biotechnology [1] and rapid transformation in a model plant system is desirable. Transformation allows researchers to analyse gene expression with reporter genes, rescue mutations with a wild type version of the gene, and take advantage of CRISPR/Cas9 for genome editing. Throughout the evolution of *Medicago truncatula* as a model system for studying legume biology, the ability to generate transgenic plants has been an important factor. Researchers have been tweaking transformation protocols since before the first suggestion of *M. truncatula* as a model by a group of French laboratories [2]. Nolan et al. [3] described a somatic embryogenesis method to regenerate *M. truncatula* from cultured leaf explants, based on a method used for *Medicago sativa*. As part of this work they noticed that only 11% of calli formed in their system from the leaves of seed-generated plants produced embryos while 93% of the calli from regenerated plants produced embryos. In 1995, a method using protoplasts isolated from this same line to generate calli was reported [4] followed by a tissue culture transformation method that was compared on the A17 ecotype and the regeneration ecotype and produced transgenic plants in 4–10 months using *Agrobacterium tumifaciens* LBA4404 as the source of transformation [5]. Further work in 1997 introduced the R108 highly regenerative ecotype [6], followed by reports of a transformation procedure using *A. tumifaciens* EHA105 that produced plants from leaf explant starting material in 3–4 months [7–9]. In this procedure 50% of the R108 embryos regenerated into complete plants. A method using the 2HA highly regenerative ecotype used by Rose [3] and *A. tumifaciens* strain AGL1 also required 4–5 months but only 24% of the explants generated transgenic plants [10]. Variations of these protocols have been used with different starting materials by numerous labs to transform legumes (reviewed in [11]) and several of the most popular variations appear in the *Medicago truncatula* Handbook [12].

Agrobacterium-mediated transformation is ecotype dependent in many plants, as ecotypes respond differently with varying transformation efficiencies (transgenic plants/initial calli formed) [13–16]. The A17 ecotype of *M. truncatula* serves as the reference accession for the genome [17] and although used widely in labs, it is considered difficult to transform [18]. The ability to directly transform the A17 ecotype is beneficial, as many forward genetic mutants were made in A17. A report of a whole plant infiltration method for transformation of A17 excited the field [19], but a second publication using the method does not exist. Most transgenic work in A17 is currently done with composite plants using *Agrobacterium rhizogenes* transformed roots [20, 21].

The R108 ecotype has a much higher transformation efficiency as compared to the A17 ecotype [6]. The R108 ecotype is derived from the same cultivar as the A17 ecotype, but as a result of multiple rounds of selection for regeneration ability in developing the ecotype, the R108 genome differs significantly from the A17 genome and that of most other *M. truncatula* ecotypes in size and sequence [22, 23]. As a result, transferring transgenes into A17 by transforming R108 followed by genetic crosses is problematic. While crosses between A17 and R108 are possible, fertility is greatly reduced, the F1 plants are pale and sickly, and although plants from the F2 appear normal, the process is lengthy. However, all of the *Tnt1* mutants publicly available, as well as a set of plants carrying reporters for subcellular localization are in the R108 background [24, 25]. To assess methods for transformation, we have chosen R108 for a direct comparison to A17 in an effort to improve A17 transformation.

**Fig. 1:**
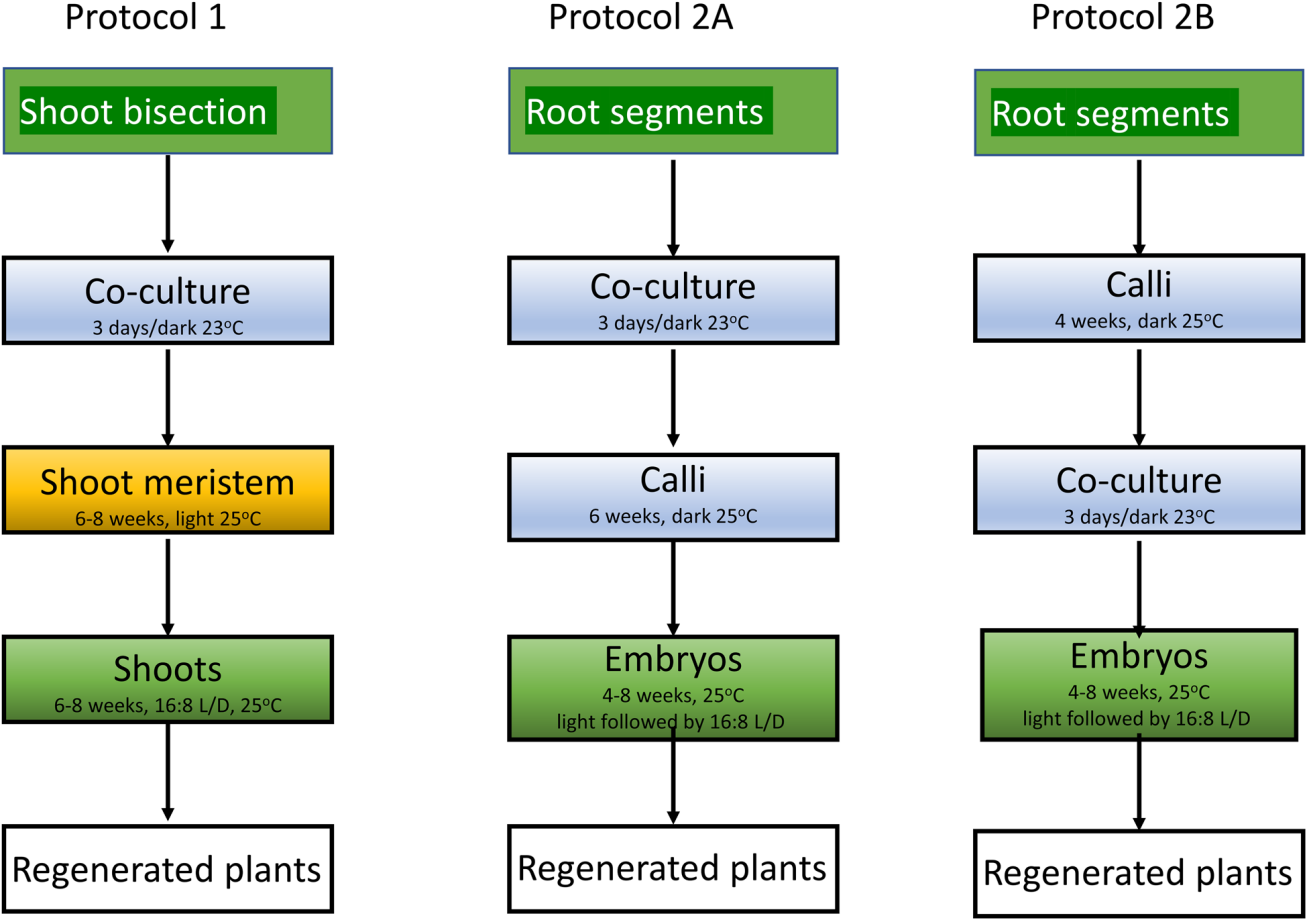
Diagram of transformation protocols explored in this work. Protocol numbers refer to sections in Materials & Methods. L/D refers to hours in Light (L) and Dark (D)

We attempted to find a new set of conditions for a shoot bisection transformation procedure used before [26] that would improve the transformation efficiency of 1% for A17 we observed when creating a plant carrying a YFP tagged protein [27]. The initial report in [26] did not mention efficiency. We replaced the temporary immersion system step with a filter paper step and compared the new shoot bisection method to methods developed in our lab from protocols for other plants, using explants from roots, with transformation both before and after induction of calli (Fig. 1). Although we were unsuccessful in finding a better method for transformation of A17, our failure to improve A17 transformation efficiency combined with a need for a large number of transgenic plants prompted us to explore reliable methods for consistent success with the R108 ecotype, and as a result transforming R108 followed by crossing to A17 is now our method of choice if it is necessary to rescue mutations or transfer reporter genes to the A17 ecotype. The constructs used in this comparison include a reporter gene construct for measuring cytokinin levels and a tagged wild type version of a gene for rescuing a mutation. We discovered a difference between time to regenerated plants and efficiency of transformation that investigators may want to consider in *M. truncatula* and the transformation of other plant species.

## Results

### Transformation of shoot bisections

#### Comparison of transformation efficiency between R108 and A17

Because the R108 genome differs significantly from the A17 genome in size and sequence [22, 23], the development of a method that allows easy transformation of A17 is desirable. When transforming A17 in our lab, the transformation efficiency was less than 1% [27], so we attempted to find a cultivation procedure that would improve the transformation efficiency, by removing steps in the protocol of [26] involving a temporary immersion system and replacing them with a co-cultivation on filter paper. The cytokinin reporter *pTCSn1::GFP*-*ER* was transformed into A17 and R108 using the newly modified shoot bisection method (Protocol 1) which included a step using filter paper to limit overgrowth of the *Agrobacterium*, a problem we encountered when using the published protocol and which the use of immersion chambers was reported to solve. The use of the same vector and protocol between A17 and R108 allowed for a direct comparison of transformation efficiencies. In two independent replicate experiments 44 of 143 explants (31%) and 36 of 99 explants (36%) of A17 showed resistance to PPT after 4 weeks on selection (Table 1). However, an additional 4 weeks later no A17 explants survived in either experiment. On the other hand, for the R108 genotype we recovered 60 of 103 (58%) and 65 of 128 (51%) resistant explants after the first 4 weeks of selection, and the numbers were not different for the following selection period (Table 1). Of these initial resistant explants 25% of the resistant explants developed to whole plants and were transferred to the greenhouse, indicating a transformation efficiency of 12–15%. Since these results showed no improvement in A17 transformation efficiency, we moved forward with comparing different methods for transforming the R108 genotype.

**Table 1:** Comparison of the shoot bisection transformation efficiency between A17 and R108

Cultivar	Experiment	Total explants	Resistant explants		Regenerated lines (lines/total explants)
			4 weeks	8 weeks	
A17	1	143	44 (31%)	0	0
	2	99	36 (36%)	0	0
R108	1	103		60 (58%)	15 (15%)
	2	128		65 (51%)	16 (12.5%)

#### Optimization of R108 transformation

Previously we had identified a *Tnt1* insertion mutant in the R108 genotype that disrupts the function of the pseudokinase CORYNE (CRN) [27]. The resulting supernodulation phenotype in *crn* mutants is dependent on the shoot phenotype of the plant [27]. The ability to rescue a mutant phenotype by transforming the mutant with a wild type copy of the gene is the canonical proof connecting a mutation with a phenotype, but because of the shoot function controlling the root phenotype [27], most hypernodulation mutants require whole plant transgenics to demonstrate rescue. The need for a whole plant transgenic versus a chimeric hairy root transformation gave us the opportunity to test multiple transformation protocols with similar constructs. Utilizing a *CRN* rescue construct described in Materials and Methods, we tested three transformation protocols on the R108 wild type, and two on the *crn* mutant in the R108 background. We transformed shoot bisections (Protocol 1) and root segments where calli were induced after transformation (Protocol 2A). Additionally, we transformed root segments after callus induction (Protocol 2B) using *pTCSn1::GFP*-*ER* in wild type R108 as our third method. A total of 120 shoot bisections were used for the transformation using Protocol 1 and 80 root segments were used with Protocol 2A. In line with our previous success transforming R108 in Table 1, 61 (50%) of the shoot bisections generated resistant explants and 36 (45%) of the root segments generated resistant explants when selected with PPT for 4 weeks. After selection, 25 plants from the shoot bisection procedure (Protocol 1) and 7 plants from the root segment procedure (Protocol 2A) developed to regenerated plants (Table 2). The time from starting plants for calli to recovering regenerated plants for transplantation to soil was 5–6 months.

**Table 2:** Comparison of transformation using different explants and vectors in the R108 ecotype

Cultivar	Material/method used for transformation	Vectors	Number of calli	Resistant explants	Regenerated plants from initial explants
R108 *crn* mutant	Shoot bisections protocol 1	*p35S:CRN:YFP*	120	61 (50%)	25 (21%)
R108 *crn* mutant	Root segments protocol 2A	*p35S:CRN:YFP*	80	36 (45%)	7 (9%)
R108	Shoot bisections protocol1	*pTCSN1*	108	55 (51%)	16 (15%)
R108	Root segments protocol 2B	*pTCSN1*	42	17 (40%)	8 (19%)

An alternate method in which the transformation of root explants occurred after the induction of calli (Protocol 2B) was compared to Protocol 1 using the *pTCSn1::GFP*-*ER* construct and R108 roots. In this case Protocol 1 resulted in 55 resistant explants from 108 calli (51%). Of those explants, 16 led to regenerated plants. In contrast, only 40% (17) of the starting calli were transformed using Protocol 2B, but 8 out of those 17 calli resulted in transformed and regenerated plants (Table 2). Both Protocol 2A and Protocol 2B required 4 months from starting plants for calli to recovering regenerated plants.

**Fig. 2:**
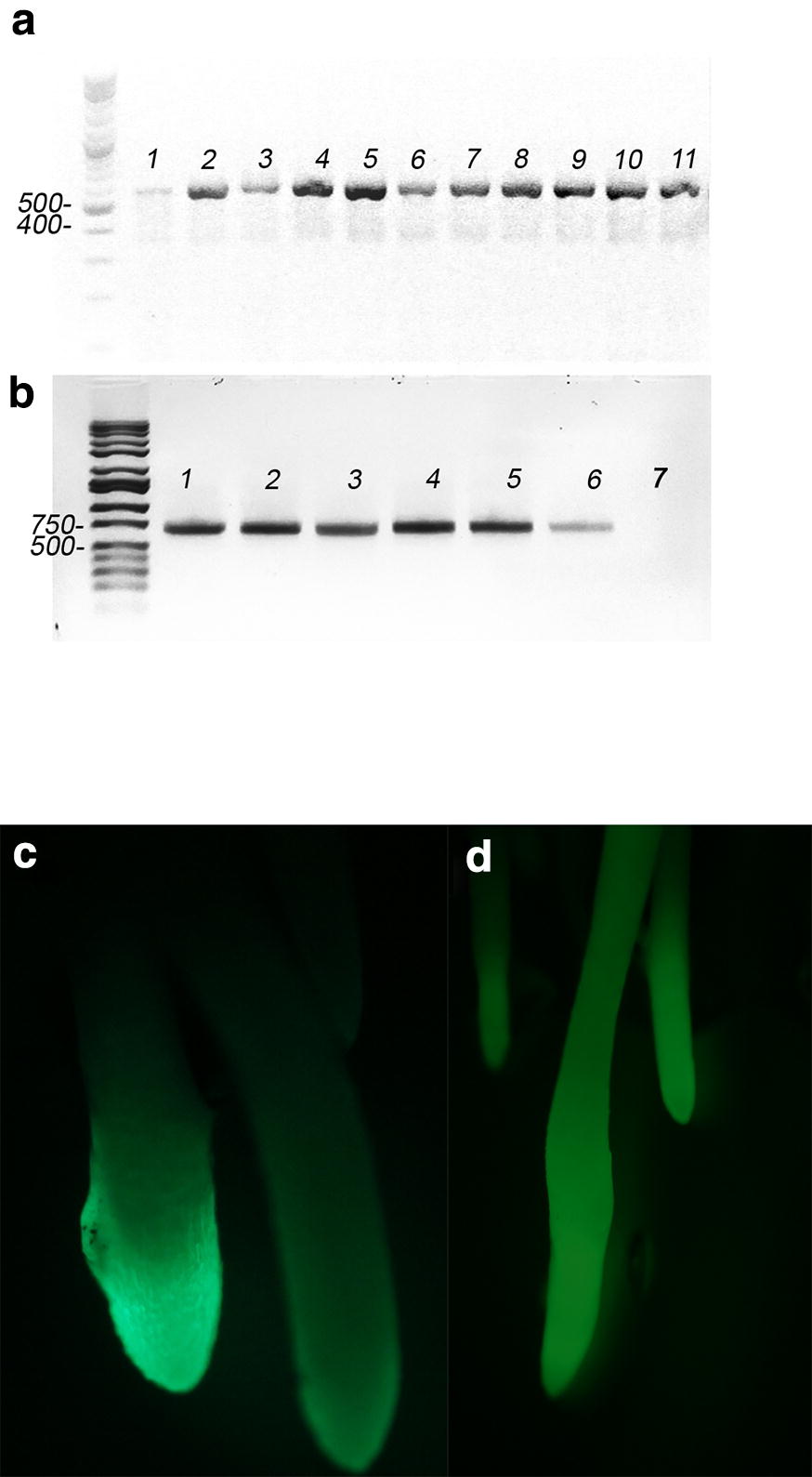
Confirmation of the transformations. **a** lanes 1-11 are PCR of a fragment of the bar gene (expected size 554 bp) using DNA from 11 individual T_0_ transformation plants of *pTCSN1::GFP*-*ER* as template. The bar gene fragment was not detected in wild type plants. **b** Lanes 1-6 are PCR amplification of a *CRN* gene fragment (expected size 526 bp) using DNA from 6 individual T_0_ transformation plants of *crn* as template. Lane 7 template uses DNA from an untransformed *crn* mutant plant. **c** Green fluorescence observed in the roots of T_0_ transformed plants of *pTCSN1::GFP*-*ER* before treatment. **d** Green fluorescence observed in in the roots of T_0_ transformed plants of *pTCSN1::GFP*-*ER* after 24 h treatment of 15 μM BAP

The regenerated plants from root explants were verified by PCR to confirm transfer of the *bar* gene for *pTCSN1* (Fig. 2a) or the *CRN* gene for *p35S:CRN:YFP* (Fig. 2b). Because the cytokinin reporter *TCSn::GFP* is sensitive to phosphorelay signaling in Arabidopsis and maize cellular assays [28], further confirmation of successful transformation of pTCSN1 was obtained by observing the expression of the GFP reporter by microscopy. Transgenic Arabidopsis *TCSn::GFP* plants exhibit strong and dynamic GFP expression patterns consistent with known cytokinin functions [29, 30], therefore we observed the expression of the GFP reporter in our plants in an area of the root undergoing rapid cell division (Fig. 2c). GFP signal was detected in this area and increased 24 h after treatment with 15 μM BAP to throughout the root (Fig. 2d). Thus, PCR and the GFP reporter results both indicate that all R108 transformations were successful.

## Discussion

Our results (Table 1) confirm that with our Protocol 1, cultivar R108 is much more efficiently transformed than the A17 cultivar. The A17 cultivar yielded fewer resistant explants indicating transformation in the early stages of the protocol, and none of the explants survived the entire procedure. In contrast, R108 consistently yielded regenerated lines from 14 to 15% of the transformed explants in multiple treatments. If the changes to the procedure had no effect on the 1% regeneration efficiency previously observed [27], the expected result of 1 transgenic plant from each trial may not have been observed simply by chance. Therefore we cannot say that the results are worse, only that there was no improvement. The cause of loss of the initially A17 resistant explants was not determined, but we speculate these were not transformation events but natural resistance.

In order to avoid chimeric plants, a problem observed in multiple species [31, 32], PPT selection was applied during the entire development of the resistant explants in the present study, and this might have resulted in inhibiting the formation and development of shoots and roots [33]. PPT can alter cell metabolism in *M. truncatula* [34], however, selection with kanamycin and hygromycin are both leaky in our hands, and while this leakiness results in more explants, those explants do not usually contain the transgene or are chimeric. All T0 lines of R108 and mutants reported in this work yielded T1 progeny containing the transgene, confirming our choice of PPT as the selection agent and transformation of the seed-bearing organs.

While all protocols took 5–6 months from plant material to regenerated plants, Protocol 2B calli can be maintained undifferentiated in culture, making it possible to use the calli for future transformations and reduce the time to regenerated plants to 4 months.

## Conclusions

The transformation efficiency of the shoot bisection protocol was higher than that of root segments by about 5% no matter what protocol or vector was used (Table 2). However, the percentage of resistant calli that led to transgenic plants was highest using root segments and the reporter gene in Protocol 2B, followed by Protocol 2A at 10% lower transformation efficiency. It should be noted that the constructs used in Protocol 2A and 2B were both fluorescent reporter genes but one was driven by a cytokinin responsive promoter and the other was fused to a native gene and driven by the 35S promoter, differences which could have affected transformation efficiency. The shoot bisection transformation (Protocol 1) required 5–6 months from start to transplantable transgenics, versus 4 months for plants from the root calli (Protocol 2B). An additional consideration for our lab was the length of the selection period during which constant monitoring is required. During this period, along with new tissue development, some older tissue dies and requires removal to maintain contact necessary for nutrient delivery to the new tissue. Explants that were co-cultivated first (Protocol 2A) developed asynchronously, requiring preparation of several media types at the same time in a single transformation. For the protocol that starts from root segment calli (Protocol 2B) the explants developed more synchronously, resulting in less labor and also less time in culture. Because root calli can be maintained undifferentiated in culture, it is possible to always have material for transformation ready to initiate. For labs performing multiple transformations for which only a few lines are needed for each transformation, the lower transformation efficiency of Protocol 2B may be offset by the higher percentage of calli that are transformed and the length of time required to obtain transgenic plants. These considerations should be useful to those planning CRISPR/Cas9 experiments in *M. truncatula* and other dicots that are regenerated through tissue culture.

## Materials and methods

### Scarification and germination of seeds

Seeds of *Medicago truncatula* genotype A17, R108 and the *crn* mutant in the R108 background were scarified in sulfuric acid for 8 min and rinsed five times with sterile water. The scarified seeds were then sterilized with a 1.5% (v/v) solution of sodium hypochlorite for 1 min and washed three times with sterile water. After soaking in sterile water and shaking at 100 rpm for 2 h in a MaxiRotator (Lab-Line Instruments Inc., USA), they were dried and stored in the dark at 4 °C overnight.

The seeds were then placed onto petri dishes containing Germination Media (GM): SH basal medium supplemented with vitamins (Phytotechnology Laboratories, USA), 2% (w/v) sucrose (Caisson Labs, USA), 13 mg L^−1^ calcium gluconate (Phytotechnology Laboratories, USA), 1.0 mg L^−1^ 6-benzylaminopurine (BAP) (Phytotechnology Laboratories) and solidified with 0.8% (w/v) plant agar (Caisson Labs, USA). After growing for 4–7 days at 25 °C, with a 16-h photoperiod, the seedlings were used as donor plants for both root and shoot explants.

### *Agrobacterium tumefaciens* strain and binary vectors

*Agrobacterium tumefaciens* strain EHA105 [35] was used in this study, first used in *M. truncatula* in [7]. The binary vector 35S:CRN-YFP/HA which carries a chimeric phosphinothricin acetyltransferase gene (*bar*) [36] under the control of CaMV 35S promoter was used for transformation. This vector was created from a pDONR vector carrying the *CRN* sequence described in [27] and cloned into pEarleygate101 via the LR reaction as described for SUNN in [27]. A second binary vector, the cytokine reporter *pTCSn 1::GFP*-*ER* [29, 30] (gift of Bruno Mueller) was also used for transformation and comparison. The vectors were introduced into *A. tumefaciens* by electroporation [37]. Single colonies of *A. tumefaciens* were cultivated on solid LB medium (1.5% agar, Becton–Dickinson and Company, USA) containing 25 mg L^−1^ kanamycin (Phytotechnology Laboratories, USA) for 2 days at 28 °C. Colonies were transferred to a second petri dish and cultured in the same condition as described above for an additional 2 days before being used in transformations.

In all protocols, media were prepared with 18.2 MΩ.cm nanopore RO water (Purelab flex, ELGA VEOLIA, USA) and pH adjusted to 5.7 or 5.4 (see Additional file 1 for details). Calli were sealed in petri-dishes (100 mm × 25 mm, VWR, USA) using medical tape (3 M Micropore, USA). Each plate was prepared with 40 mL of media. Artificial light conditions are 244 lm/m^2^ provided by F40 T12 Plant and Aquarium bulbs (General Electric, USA) 28 cM from the plates.

### Protocol 1: Infection of shoot bisection explants by *A. tumefaciens* selection and plant regeneration

**Fig. 3:**
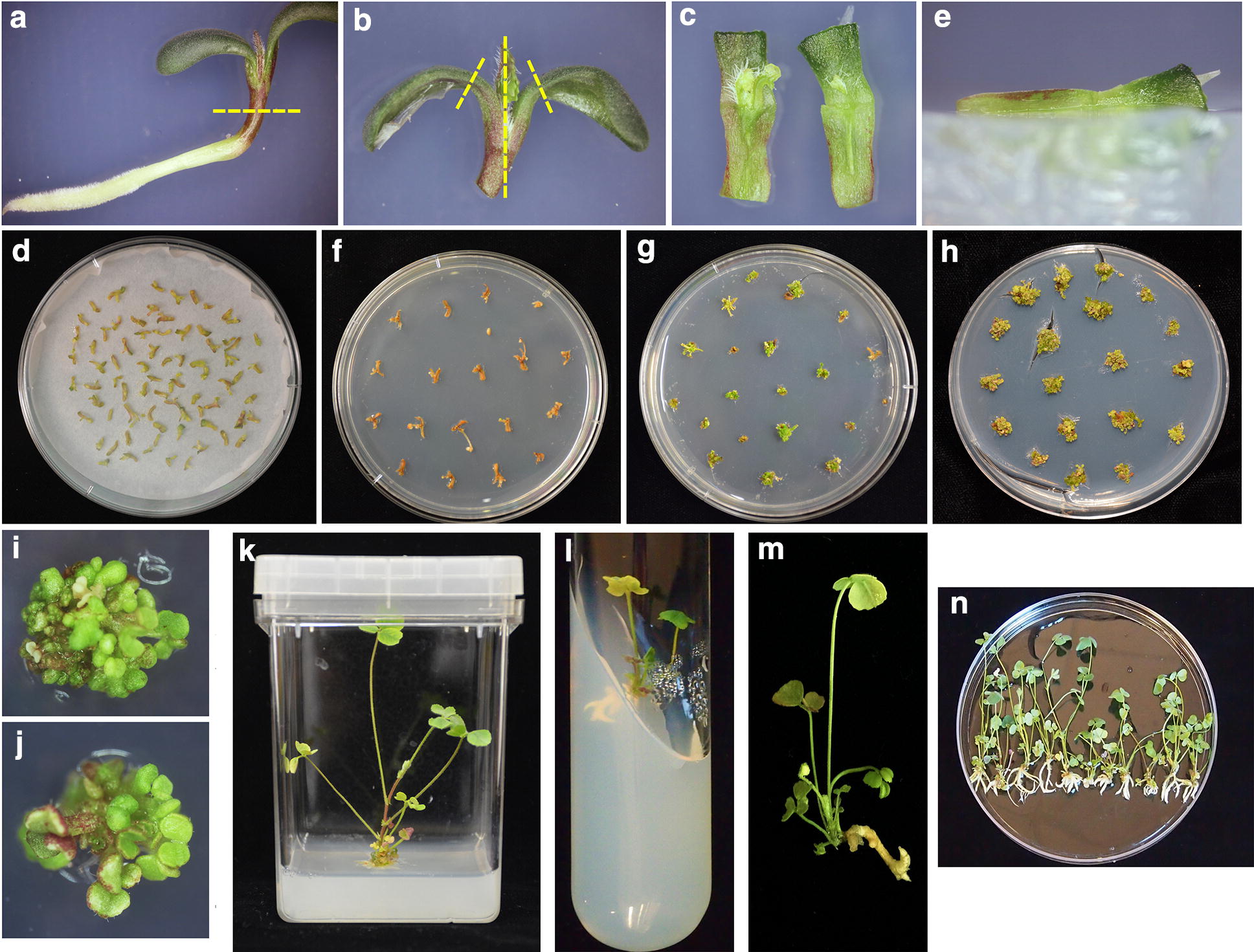
Steps in Tissue Culture Protocol 1. **a** Initial cut on 4 day old seedling. **b** Bisected shoot starting material (cotyledons cut off). **c** Shoot bisection starting material. **d** Co-cultivation with filter paper on media. **e** Inserting half of explant into agar so the meristem contacts the PPT medium. **f** Untransformed controls and **g** Explants transformed with construct after 2 rounds of selection of 15 days each. **h** Transfer to shoot development media. **I**, **J** Shoots arising from **h**. **k** Intact plants in magenta box or **l** Test tube. **m**, **n** Regenerated plants ready for transfer into soil

*A. tumefaciens* colonies from the above media were mixed thoroughly and cultured in 30 mL of suspension media (STM1-Additional file 1) for 1 h to OD 600 = 0.5. Following the preparation procedure of [27], developing shoots of R108 and *crn* mutants were bisected (Fig. 3a–c). The bisected shoots were inoculated with the *A. tumefaciens* suspension above and shaken at 100 rpm for 20 min (MAXQ 4450, Thermo Scientific, USA). After blot-drying the explants with sterile filter paper, the explants were placed separately on filter paper on the surface of the co-cultivation media (STM2 media-Additional file 1) and cultured in the dark for 3 days at 23 °C, arranging the explants so they did not touch and the plates were not stacked (Fig. 3d). Afterwards, the explants were transferred onto selection media (STM3 media-Additional file 1). One half of the side of the explant was inserted into the media to insure the meristem contacted the media and at the same time allowed the untransformed cells to be exposed to the selection agent (Fig. 3e), and the plates were transferred to artificial light at 25 °C until shoot elongation.

The media was changed every 2 weeks, and after 2 rounds of selection (Fig. 3f, g), the resistant explants were then transferred onto shoot propagation media (STM4-Additional file 1) modified from STM3 media by lowering concentration of BAP and removing the NAA, and exposed to a 16/8 light/dark cycle under artificial light at 25 °C. Two to 4 weeks after being transferred to STM4 media (Fig. 3h), resistant explants (identified by green growth in Fig. 3i, j) were transferred onto shoot development media (STM5-Additional file 1) modified from STM4 media by changing BAP to 0.5 mg L^−1^, and the explants remained on this media 6-8 weeks. When shoots developed and small leaves were formed (Fig. 3d), the explants were transferred to root development media (STM6-Additional file 1). About 4–6 weeks after being transferred onto STM6 media, the plantlets developed roots and were transferred to magenta boxes (Fig. 3k) or 25 mm × 200 mm glass tubes (Fig. 3l). Upon development of a lateral root (Fig. 3m) the transgenic plants were transferred into soil (Fig. 3n) and grown up to flowering stage in a greenhouse.

### Protocol 2A *A. tumefaciens*-mediated transformation before induction of calli from root segments of R108 *crn* mutants

**Fig. 4:**
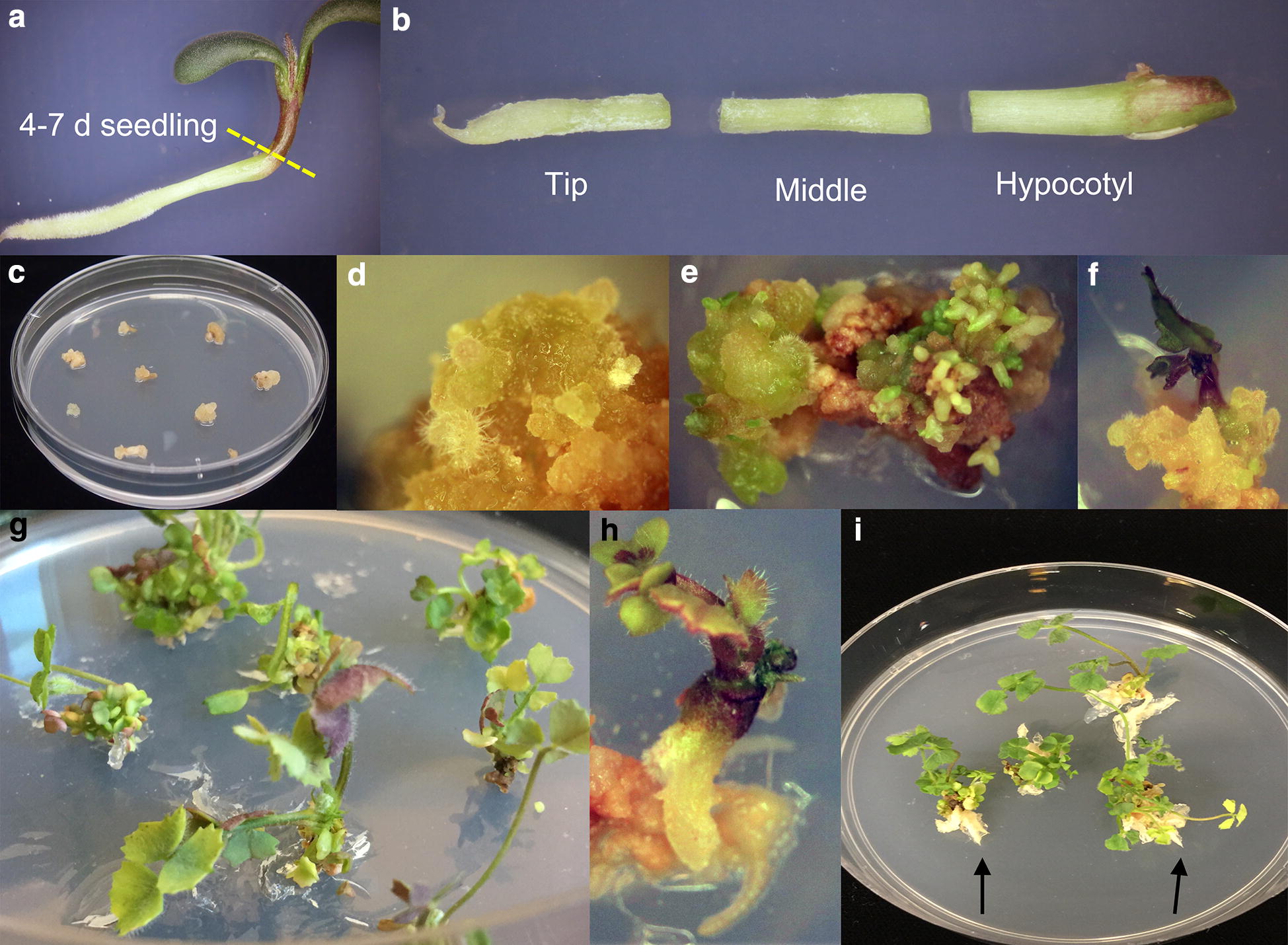
Steps in Tissue Culture Protocol 2A/B. **a** Initial seedlings used as source. **b** Dissection of root into segments. **c** Calli induced after 4 or 6 weeks on CTM3 or RCTM3. **d** Calli after 1 week on RCTM4. **e** Embryos forming after 4 weeks on RCTM4. **f** Shoot forming after 1 week on RCTM5. **g**, **h** Shoot developed and root started to differentiate after 4 weeks on RCTM5. **i** Root (arrow) developing after 3 weeks on RCTM6

*A. tumefaciens* carrying the *crn* vector were cultured for 1 h in 30 mL of suspension media (RCTM1-Additional file 1), modified from MTR-1 media [38]. Roots of 4–7 days seedlings of R108 *crn* mutants (see scarification and germination of seeds) were cut into 3 segments (Fig. 4a, b), discarding the root tips as we were unable to recover de-differentiated calli from root tips in preliminary experiments. The segments were suspended in liquid RCTM1 media with *A. tumefaciens* (OD 600 = 0.5) and shaken at 100 rpm for 20 min. The explants were then blot-dried with sterile filter paper and placed on filter paper on the surface of the co-cultivation media (RCTM2-Additional file 1). The explants were placed so that they did not touch each other and incubated for 3 days at 23 °C in the dark.

The calli were then transferred for selection to RCTM3 media (Additional file 1) modified from MTR-2 media [38] and incubated in the dark at 25 °C. The calli were selected for 6 weeks, changing the media every 2 weeks (Fig. 4c). Note that waiting to add PPT to RCTM3 until the first change of media may give better results. The resistant calli at the end of 6 weeks were transferred onto shoot development media RCTM4 (Additional file 1) and moved to artificial light at 25 °C for the formation of zones of embryogenesis (Fig. 4d). RCTM4 media is modified from MTR-3 media described in [38]. After 2-4 weeks on RCTM4 in the light some clearly visible embryos developed (Fig. 4e, f) and the explants were transferred to RCTM5 media (Additional file 1) under a 16/8 light/dark cycle for further shoot development and root differentiation (Fig. 4g). Another 2–4 weeks later, they were transferred onto RCTM6 media (Additional file 1) for root development (Fig. 4h). A final 2–4 weeks later, the roots of the transgenic plants developed (Fig. 4i), and they were transferred into soil and grown out to flowering stage in a greenhouse.

### Protocol 2B: *A. tumefaciens*-mediated transformation after induction of calli from root segments of R108

Roots of 4–7 days seedlings of R108 were cut into 2–3 segments and transferred onto callus inducing media (CIM1-Additional file 1) modified from [38] and cultured in the dark at 25 °C for 4 weeks, changing the media every 2 weeks. After 4 weeks the formed calli were induced and utilized for transformation. The R108 calli were suspended and co-cultivated with *A. tumefaciens* in RCTM1 and RCTM2 media as described as above. After being co-cultivated in the dark for 3 days on RCTM2, the calli were transferred to RCTM3 media and incubated in artificial light for selection and seedling development, changing the media every 2 weeks. The seedling development and the root formation procedure were the same as described for the resistant calli in the *crn* transformation. The resistant seedlings of the *pTCSn1::GFP*-*ER* and the *crn* transformation were transferred to root development media RCTM6 for whole plant development at 25 in a 16/8 Light/Dark cycle. After 2–3 weeks, they were transferred to soil and grown out to flowering stage in a greenhouse.

### DNA extraction and PCR analyses

DNA from individual plants was extracted using the FTA DNA extraction kit (Whatman, GE Healthcare). Leaves of individual plants were pressed onto the FTA paper, and DNA extracted following the manufacturer’s instructions.

The *bar* and *crn* transgenes were amplified in transgenic plants by PCR using primers shown in Additional file 2. PCR reactions consisted of 1 × Go Taq™ PCR reaction buffer (Promega, USA), 2 μM dNTPs, 2 μM primers, and 3 U GoTaq™ polymerase in a volume of 10 μL. Reaction conditions were 95 °C 2 min, followed by 40 cycles of 95 °C 10 s, 55 °C 10 s, and 72 °C 30 s, followed by a 2 min hold at 72 °C. The expected PCR products were 554 bp for *bar* and 526 bp for *crn*.

### Detection of GFP

Fluorescence microscopy images of plant segments expressing GFP were collected using an Olympus SZX12 microscope and photographed with a DP73 digital camera (Olympus, Japan). Expression response of the cytokinin reporter construct in roots of *pTCSn1::GFP*-*ER* transformed T_0_ plants was observed by incubating the plants in 15 μM BAP, and fluorescence observed before treatment and after treatment at 1 h and 24 h as in [29].

## Additional files

**Additional file 1.** Media recipes.

**Additional file 2.** Primers used in this work.

## Abbreviations

BAP: 6-benzylaminopurine
NAA: naphthaleneacetic acid
2,4-d: dichlorophenoxyacetic acid
PPT: phosphinothricin
