# Correction to: Comparison of efficiency and time to regeneration of *Agrobacterium*-mediated transformation methods in *Medicago truncatula*

**DOI:** 10.1186/s13007-019-0480-2

**Published:** 2019-08-19

**Authors:** Li Wen, Yuanling Chen, Elise Schnabel, Ashley Crook, Julia Frugoli

**Affiliations:** 1 Department of Genetics and Biochemistry, Clemson University, Clemson, USA; 2 Department of Food and Biological Engineering, Changsha University of Science and Technology, Changsha, People’s Republic of China; 3 College of Life Sciences, South China Agricultural University, Guangzhou, People’s Republic of China; 4 Department of Biology, University of North Carolina at Chapel Hill, Chapel Hill, NC 27599 USA

## Correction to: Plant Methods (2019) 15:20 10.1186/s13007-019-0404-1

Unfortunately, the original version of the article [1] contained an error. It has been brought to the attention of authors that the R108 ecotype was not derived from A17 but is rather a different subspecies derived from an accession collected in Israel, with a different plastid genome, which likely affects the ability to cross to A17.

Under the Background section, third paragraph, the second to fourth sentences should read as “The R108 ecotype is derived from a population in Israel and is a different subspecies from A17 [2] the R108 genome differs significantly from the A17 genome and that of most other *M. truncatula* ecotypes in size and sequence [22, 23]. As a result, transferring transgenes into A17 by transforming R108 followed by genetic crosses is problematic. While crosses between A17 and R108 are possible, fertility is greatly reduced, the F1 plants are pale and sickly likely due to differences in the plastid genome [3], and although plants from the F2 appear normal, the process is lengthy. However, all of the *Tnt1* mutants publicly available, as well as a set of plants carrying reporters for subcellular localization are in the R108 background [24, 25].”

The two new references [2, 3] are given below:
