# Supplementary material for: Comparison of efficiency and time to regeneration of Agrobacterium-mediated transformation methods in Medicago truncatula
Source: Plant Methods. 2019 Feb 28;15:20. doi: 10.1186/s13007-019-0404-1 (PMC6394069; doi:10.1186/s13007-019-0404-1)
Supplement: Supplementary file 1 — Additional file 1. Media recipes. [file 13007_2019_404_MOESM1_ESM.docx]

**Additional file 1.Media Recipes**

1. **Germination Media (GM)**

| **Chemical** | **100 ml** |
| --- | --- |
| SH basal medium (Phytotechnology Labs) | 320 mg |
| MS Vitamins 1000X  (Phytotechnology Labs) | 100 μl |
| Sucrose (Caisson Labs) | 2 g |
| Calcium gluconate (Phytotechnology Labs) | 130 mg |
| pH to | 5.7 |
| Plant Agar (Caisson Labs) | 0.8 g |
| BAP stock solution (3.4 mg/ml) (prepared from Phytotechnology Labs) | 30 μl |

1. **Suspension Transformation Media (STM) Protocol 1**

| **Chemical** | **STM1**  **100 ml** | **STM2**  **100 ml** | **STM3**  **100 ml** | **STM4**  **100 ml** | **STM5**  **100 ml** | **STM6**  **100 ml** |
| --- | --- | --- | --- | --- | --- | --- |
| SH basal medium  (Phytotechnology Labs) | 320 mg | 320 mg | 320 mg | 320 mg | 320 mg | 320 mg |
| MS Vitamins 1000X  (Phytotechnology Labs) | 100 μl | 100 μl | 100 μl | 100 μl | 100 μl | 100 μl |
| Sucrose (Caisson Labs) | 2 g | 2 g | 2 g | 3 g | 3 g | 1 g |
| Calcium gluconate  (Phytotechnology Labs) | 130 mg | 130 mg | 130 mg | 130 mg | 130 mg | 130 mg |
| Casein peptone (Alfa Aesar) | 200 mg | 50 mg | 0 | 0 | 0 | 0 |
| Inositol  (Becton Dickinson and Company) | 20 mg | 20 mg | 0 | 0 | 0 | 0 |
| pH | 5.4 | 5.4 | 5.7 | 5.7 | 5.7 | 5.7 |
| Acetosyringone stock solution  (100 mM) (prepared from Phytotechnology Labs) | 100 μl | 100 μl | 0 | 0 | 0 | 0 |
| Filtered Glucose (Dextrose) stock solution 30g/ml  (Phytotechnology Labs) | 10 ml | 3.3 ml | 0 | 0 | 0 | 0 |
| Plant Agar (Caisson Labs) | 0 | 0.8 g | 0.8 g | 0.8 g | 0.8 g | 0.8 g |
| BAP stock solution (3.4 mg/ml) (prepared from Phytotechnology Labs) | 0 | 30 μl | 100 μl | 58.8 μl | 14.7 μl | 0 |
| Cefotaxime stock solution (250 mg/ml) (prepared from Caisson Labs) | 0 | 0 | 100 μl | 100 μl | 100 μl | 100 μl |
| Carbenicillin stock solution (400 mg/ml) (prepared from Caisson Labs) | 0 | 0 | 100 μl | 100 μl | 100 μl | 100 μl |
| Timentin stock solution (100 mg/ml) (prepared from Phytotechnology Laboratories) | 0 | 0 | 100 μl | 100 μl | 100 μl | 100 μl |
| PPT stock solution (2 mg/ml) (prepared from Phytotechnology Labs) | 0 | 0 | 100 μl | 100 μl | 100 μl | 100 μl |
| NAA stock solution (1 mg/ml) (prepared from Phytotechnology Laboratories) | 0 | 0 | 10 μl | 0 | 0 | 20 μl |

1. **Root Callus Transformation Media (RCTM) Protocol 2A & 2B**

| **Chemical** | **RCTM1**  **100 ml** | **RCTM2**  **100 ml** | **RCTM3**  **100 ml** | **RCTM4**  **100 ml** | **RCTM5**  **100 ml** | **RCTM 6**  **100 ml** |
| --- | --- | --- | --- | --- | --- | --- |
| MS Basal media  (Caisson Labs) | 433 mg | 433 mg | 433 mg | 433 mg | 0 | 0 |
| SH basal medium (Phytotechnology Labs) | 0 | 0 | 0 | 0 | 320 mg | 320 mg |
| MS Vitamins 1000X  (Phytotechnology Labs) | 100 μl | 100 μl | 100 μl | 100 μl | 100 μl | 100 μl |
| Sucrose (Caisson Labs) | 3 g | 3 g | 3 g | 2 g | 2 g | 1 g |
| Calcium gluconate (Phytotechnology Labs) | 0 | 0 | 0 | 0 | 130 mg | 130 mg |
| Acetosyringone stock solution  (100 mM) (prepared from Phytotechnology Labs) | 100 μl | 100 μl | 0 | 0 | 0 | 0 |
| pH | 5.7 | 5.7 | 5.7 | 5.7 | 5.7 | 5.7 |
| Plant Agar (Caisson Labs) | 0 | 0.8 g | 0.8 g | 0.8 g | 0.8 g | 0.8 g |
| 2,4-D stock solution (5 mg/ml)  (prepared from Phytotechnology Labs) | 100 μl | 100 μl | 100 μl | 0 | 0 | 0 |
| BAP stock solution (3.4 mg/ml) (prepared from Phytotechnology Labs) | 14.7 μl | 14.7 μl | 14.7 μl | 14.7 μl | 14.7 μl | 0 |
| PPT stock solution (2 mg/ml) (prepared from Phytotechnology Labs) | 0 | 0 | 250 μl | 250 μl | 100 μl | 0 |
| Timentin stock solution (100 mg/ml) (prepared from Phytotechnology Laboratories) | 0 | 0 | 100 μl | 100 μl | 0 | 0 |
| Cefotaxime stock solution (250 mg/ml) (prepared from Caisson Labs) | 0 | 0 | 100 μl | 100 μl | 100 μl | 100 μl |
| NAA stock solution (1 mg/ml) (prepared from Phytotechnology Labs) | 0 | 0 | 0 | 0 | 0 | 20 μl |

1. **Callus Inducting Media (CIM1) Protocol 2B**

| **Chemical** | **CIM1**  **100 ul** |
| --- | --- |
| MS Basal media (Caisson Labs) | 320 mg |
| MS Vitamins 1000X (Phytotechnology Labs) | 100 μl |
| Sucrose (Caisson Labs) | 3 g |
| pH | 5.7 |
| Plant Agar (Caisson Labs) | 0.8 g |
| 2,4-D stock solution (5 mg/ml)  (prepared from Phytotechnology Labs) | 100 μl |
| BAP stock solution (3.4 mg/ml)  (prepared from Phytotechnology Labs) | 14.7 μl |
