# Supplementary material for: Comparison of efficiency and time to regeneration of Agrobacterium-mediated transformation methods in Medicago truncatula
Source: Plant Methods. 2019 Feb 28;15:20. doi: 10.1186/s13007-019-0404-1 (PMC6394069; doi:10.1186/s13007-019-0404-1)
Supplement: Supplementary file 2 — Additional file 2. Primers used in this work. [file 13007_2019_404_MOESM2_ESM.docx]

**Additional file 2. Primers used***

| Primer name | Sequence 5’ to 3’ |
| --- | --- |
| BAR-F | cctccctcgagATGAGCCCAGAACGACGCCC |
| BAR-R | cctccctcgagTCAGATCTCGGTGACGGGCA |
| CRNwt-F | TTCGCCGGAGAGTAGGAAAAAGTC |
| CRNwt-R | TGTAATCGCATGTGTCAGTATC |

*Lower case indicates extensions used to create restriction sites not used in this work.
